# Association between modified frailty index and postoperative delirium in patients after cardiac surgery: A cohort study of 2080 older adults

**DOI:** 10.1111/cns.14762

**Published:** 2024-06-25

**Authors:** Hongtao Cheng, Yitong Ling, Qiugui Li, Xinya Li, Yonglan Tang, Jiayu Guo, Jing Li, Zichen Wang, Wai‐kit Ming, Jun Lyu

**Affiliations:** ^1^ School of Nursing Jinan University Guangzhou China; ^2^ Department of Clinical Research The First Affiliated Hospital of Jinan University Guangzhou China; ^3^ Department of Neurology The First Affiliated Hospital of Jinan University Guangzhou China; ^4^ School of Public Health Shanxi University of Chinese Medicine Xianyang China; ^5^ Department of Infectious Diseases and Public Health City University of Hong Kong Hong Kong China; ^6^ Guangdong Provincial Key Laboratory of Traditional Chinese Medicine Informatization Guangzhou China

**Keywords:** cardiac surgery, frailty, modified frailty index, older adults, postoperative delirium, pressure injury

## Abstract

**Aim:**

To evaluate the association between frailty and postoperative delirium (POD) in elderly cardiac surgery patients.

**Methods:**

A retrospective study was conducted of older patients admitted to the intensive care unit after cardiac surgery at a tertiary academic medical center in Boston from 2008 to 2019. Frailty was measured using the Modified Frailty Index (MFI), which categorized patients into frail (MFI ≥3) and non‐frail (MFI = 0–2) groups. Delirium was identified using the confusion assessment method for the intensive care unit and nursing notes. Logistic regression models were used to examine the association between frailty and POD, and odds ratios (OR) with 95% confidence intervals (CI) were calculated.

**Results:**

Of the 2080 patients included (median age approximately 74 years, 30.9% female), 614 were frail and 1466 were non‐frail. The incidence of delirium was significantly higher in the frail group (29.2% vs. 16.4%, *p* < 0.05). After adjustment for age, sex, race, marital status, Acute Physiology Score III (APSIII), sequential organ failure assessment (SOFA), albumin, creatinine, hemoglobin, white blood cell count, type of surgery, alcohol use, smoking, cerebrovascular disease, use of benzodiazepines, and mechanical ventilation, multivariate logistic regression indicated a significantly increased risk of delirium in frail patients (adjusted OR: 1.61, 95% CI: 1.23–2.10, *p* < 0.001, *E*‐value: 1.85).

**Conclusions:**

Frailty is an independent risk factor for POD in older patients after cardiac surgery. Further research should focus on frailty assessment and tailored interventions to improve outcomes.

## Graphical abstract

This study showed a statistically significant association between higher frailty, as assessed by the MFI, and an increased risk of postoperative delirium in older cardiac surgery patients. In order to improve outcomes in this vulnerable population, the need for personalized intervention strategies was highlighted.

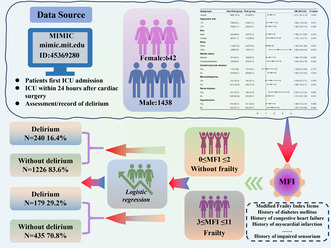

## 1 INTRODUCTION

Delirium is an acute encephalopathy syndrome characterized by inattention and global cognitive dysfunction.^1^ In patients undergoing cardiac surgery, it is a common complication during their stay in the ICU, with an incidence rate ranging from 3% to 56%.^2^, ^3^ The incidence of POD is approximately four times higher in elderly patients compared to younger patients.^4^ Such events are associated with adverse outcomes, including prolonged hospital stays, increased hospital costs, increased mortality, and significant cognitive impairment.^5^ Some preventive measures can reduce the incidence of POD.^6^ Therefore, early identification of patients at risk for delirium and implementation of these preventive strategies are critical.

Frailty is a multidimensional geriatric syndrome characterized by a loss of physiological reserves that leads to an increased susceptibility to adverse outcomes.^7^ In elderly surgical patients, its prevalence is believed to be even higher, leading to adverse health outcomes including falls, emergency department visits, hospital admissions, disability, various complications, and even death.^8^, ^9^

Frail individuals often present with cognitive decline, and stress can exacerbate cerebral dysfunction, leading to delirium. Evidence suggests that both frailty and delirium result from an imbalance in multiple physiological systems and homeostasis, involving various pathophysiological pathways such as inflammation, atherosclerosis, and nutritional deficiencies.^10^ Both frailty and delirium have been found to be independently associated with poor health outcomes.^11^, ^12^ In addition, frailty is correlated with a 2‐ to 6‐fold increased risk of delirium, which may influence the association between frailty and mortality.^13^, ^14^ Previous research has suggested that in surgical patients, frailty may be a more accurate predictor of perioperative complications and mortality than actual age.^15^, ^16^, ^17^, ^18^ While some studies have associated frailty with POD, others have not.^19^, ^20^ However, frailty and POD share certain risk factors, such as aging, that may confound their association.^21^

Studies show that advanced age is associated with a higher risk of adverse medical outcomes, and age‐related factors increase the vulnerability and frailty of the elderly.^21^, ^22^, ^23^ Particularly in the ICU setting, elderly patients are at increased risk of developing delirium, which is further correlated with increased hospital and ICU length of stay and 90‐day mortality.^24^ Frailty also plays a critical role in the outcomes of elderly patients in the ICU.^25^ However, the association between frailty and delirium, which may pose significant challenges to elderly cardiac surgery patients, has not been thoroughly investigated. Therefore, the purpose of this study is to further investigate whether frailty is associated with the occurrence of POD in elderly cardiac surgery patients. Furthermore, the primary hypothesis of the study is that among older cardiac surgery patients, frail individuals are at higher risk for POD.

## 2 METHODS

### 2.1 Data source and patients

This was a retrospective observational study using data from the Medical Information Mart for Intensive Care IV (MIMIC‐IV) database.^26^, ^27^ MIMIC‐IV is an open and freely accessible database containing detailed clinical data on tens of thousands of patients from Beth Israel Deaconess Medical Center in Boston, Massachusetts, from 2008 to 2019. This database integrates multiple electronic medical records, providing researchers with rich information on patient demographics, vital signs, laboratory test results, and diagnoses based on International Classification of Diseases, Ninth Revision (ICD‐9) and Tenth Revision (ICD‐10) codes.^28^ Because the data in this database are de‐identified, it is not necessary to obtain informed consent from patients for this study. To ensure appropriate access and use of this database, one of the authors on our team has completed the required training and secured certification to access the database (Record ID: 45369280).

**FIGURE 1:**
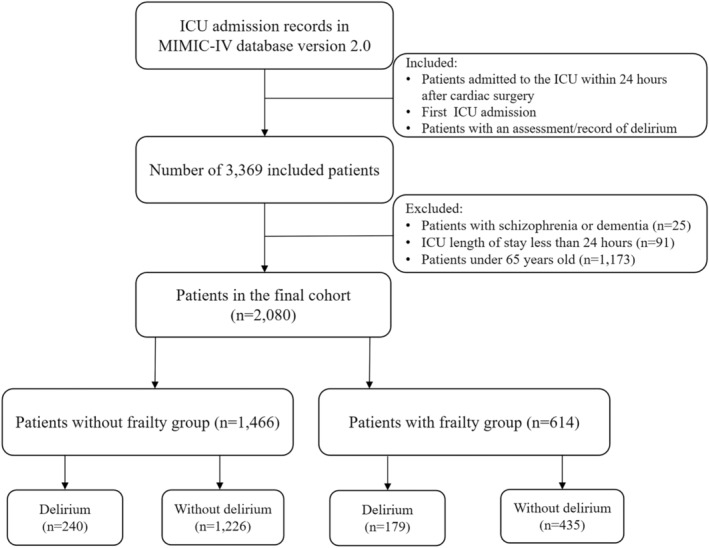
Study inclusion and exclusion flowchart. ICU, intensive care unit; MIMIC‐IV, Medical Information Mart for Intensive Care IV.

Patients were included if they met the following criteria: (1) patients admitted to the ICU within 24 h of cardiac surgery; (2) patients admitted to the ICU for the first time; (3) patients with delirium assessment/record. To identify patients who underwent cardiac surgery, we used ICD‐9 and ICD‐10 codes,^29^ a list of which is detailed in Table S1. These groups were identified using the “procedures_icd” table in the MIMIC‐IV database. We excluded patients who (1) had dementia or schizophrenia, (2) had an ICU stay of less than 24 h, and (3) were younger than 65 years. After applying the above screening criteria, we obtained 2080 eligible elderly patients after cardiac surgery for subsequent research analysis. The patient screening process is described in detail in Figure 1.

### 2.2 Data extraction

The study used structured query language to extract data. The following data were extracted including (1) general patient information; (2) lifestyle habits; (3) scores; (4) vital signs; (5) laboratory tests; (6) comorbidities; (7) mechanical ventilation, surgery type [coronary artery bypass grafting (CABG), valve surgery, combined mitral and aortic valve replacement, and combined cardiac surgery]; (8) adverse outcomes. Of note, vital signs, laboratory tests, and data for illness severity scores were extracted from the first measurement on the day of ICU admission. Comorbidities were identified using ICD‐9 and ICD‐10 codes. In addition, when considering mechanical ventilation, we specifically extracted whether the patient was receiving mechanical ventilation before the onset of delirium to ensure that mechanical ventilation was accurately considered as a potential risk factor in the statistical analysis. The missing rate of all variables in this study did not exceed 20%. For variables with a missing rate of less than 20%, we used the “mice” package, which uses the non‐missing variables for training and the random forest method for multiple imputation, to accurately estimate and impute these data.^30^ Details of the missing data in the dataset for this study are shown in Figure S1.

### 2.3 Exposure (frailty assessment)

The primary focus of this cohort study was frailty as defined by the MFI.^31^ The MFI is a scoring system consisting of 11 sub‐items that collectively reflect a patient's physical function, comorbidities, or physiological deficits. Detailed items and their construction are listed in Table S2. To obtain relevant diagnostic information from the MIMIC‐IV database, both ICD‐9 and ICD‐10 versions of the ICD codes were used. Based on the research by Hao et al. and using the general equivalence mappings provided by the Centers for Medicare and Medicaid Services (https://www.cms.gov), ICD‐10 codes in the MFI were mapped to ICD‐9 codes.^32^ These ICD‐9 and ICD‐10 codes were then used to retrieve relevant MFI items from the database. Each subitem of the MFI score was assigned a value of 1, resulting in a total score ranging from 0 to 11. Based on the MFI score, patients were classified into three categories: non‐frail (MFI = 0), pre‐frail (MFI = 1–2), and frail (MFI ≥3).^33^ To simplify the categorization of frailty status in statistical analyses and to improve the interpretability of results, the categories “pre‐frail” and “non‐frail” were combined and referred to as “non‐frail”, following the work of Tan et al.^34^

### 2.4 Outcomes and definitions

The outcomes of interest in the study were delirium and pressure injury, with delirium being the primary outcome. Delirium was assessed by healthcare professionals using the confusion assessment method for the intensive care unit (CAM‐ICU). The CAM‐ICU is one of the most widely used tools for diagnosing delirium among non‐psychiatric staff, preferred for its speed, efficiency, and reliability.^35^ A bivariate meta‐analysis reported that the method has a high sensitivity (85%) and specificity (95%).^36^ In addition, nursing notes were used as an adjunct diagnostic measure to increase the accuracy and comprehensiveness of the diagnosis. Searching for delirium‐related keywords such as “delirium,” “confusion,” “agitation,” and “altered mental status” identified additional potential cases of delirium in patients.^37^ This information was accessible through the “chartevents” table (itemid = 220,001). The median time of onset of delirium in the study patients was approximately 2 days, calculated from the start of ICU admission. Pressure injuries were determined by direct observation by nurses and categorized as stage I to IV, unstageable, and suspected deep tissue injury.^38^

### 2.5 Covariates

Covariates are variables that may be associated with the outcome but are not the primary focus of the study in statistical analyses. In selecting covariates for this study, we considered evidence from previous literature, expertise in the specific area, and data availability.^29^, ^32^ To rigorously control for potential confounders, our analysis for delirium included multivariate adjustments. These included age, sex, race, marital status, Acute Physiology Score III (APSIII), sequential organ failure assessment (SOFA), albumin, creatinine, hemoglobin, white blood cell count, type of surgery, alcohol use, smoking, cerebrovascular disease, use of benzodiazepines during ICU stay, and mechanical ventilation. In evaluating the association between frailty and pressure injury, we specifically included the Braden score, a widely accepted tool for assessing a patient's skin condition, in addition to standard demographics (age, sex, race, marital status), severity of illness scores (APSIII, SOFA), and surgery type.

### 2.6 Statistical analysis

This study followed the recommendations of STROBE (Strengthening the Reporting of Observational Studies in Epidemiology). We first performed descriptive statistical analyses of the data, comparing differences between the two groups according to frailty status. The Shapiro–Wilk test was used to assess the distribution of the continuous variables, which showed that they were all non‐normally distributed. For continuous variables that did not meet the assumption of normal distribution, we expressed them as medians with interquartile ranges and used the Wilcoxon rank‐sum test or Mann–Whitney U test for between‐group comparisons. Categorical variables were presented as frequencies and percentages, with the chi‐squared test used for group differences. When analyzing the association between frailty and study outcomes, we used logistic regression techniques to calculate odds ratios (OR) and their 95% confidence intervals (CI). MFI was treated as both a continuous variable and a dichotomous outcome. To avoid multicollinearity in our model, we used the variance inflation factor (VIF) to assess collinearity among variables. The results showed that all VIF values were less than 4, confirming no apparent multicollinearity in the model. We also visualized the relationship between MFI and delirium using restricted cubic splines. Finally, we performed subgroup analyses to examine the potential impact of variables such as age, sex, race, marital status, cerebrovascular disease, use of benzodiazepines, renal disease, and hypertension on outcomes.

Statistical analyses were performed using R software (version 4.3.0, https://www.r‐project.org/). In this study, a *p*‐value of less than 0.05 was considered statistically significant.

### 2.7 Sensitivity analysis

To ensure the robustness and stability of our study results, we first used the propensity score matching (PSM) method.^39^ Based on the aforementioned covariates, we used a logistic regression model to estimate patients' propensity scores and matched them using a 1:1 optimal matching algorithm. After matching, we calculated the standardized mean differences (SMD) of all covariates to verify the balance, with an SMD value below 0.1 typically indicating good balance between the groups. Considering that patients who die in the hospital often have more severe illnesses and those undergoing combined cardiac surgery may have more complex procedures, and given the prevalence of sepsis in the ICU and its adverse impact on patient outcomes, these factors could all confound the risk of delirium. Therefore, we performed specific analyses on three different patient groups: patients who survived to discharge (*n* = 2059), patients who underwent combined cardiac surgery (*n* = 474), and patients with sepsis (*n* = 1109). This was done to more accurately assess the risk of delirium in these specific scenarios. In addition, we excluded patients with missing data and used the full dataset for sensitivity analyses to validate the robustness of our results. We also conducted sensitivity analyses with different combinations of covariates to assess the association between frailty and delirium. Model 1 included baseline demographic information; Model 2 added severity of illness scores and laboratory parameters to Model 1; Model 3 added types of surgery, treatments and medications, and smoking and alcohol use; Model 4 added comorbidities based on Model 3. Finally, to quantify the potential influence of unmeasured confounders on the primary outcome, we assessed the *E*‐value, a tool that helps determine whether observed associations could be fully explained by unmeasured confounding.^40^ In general, a higher *E*‐value indicates greater confidence in the study results. An online web‐based calculator (https://www.evalue‐calculator.com/evalue/) was used in the study to calculate the *E*‐value.

## 3 RESULTS

### 3.1 Baseline characteristics of the participants

A total of 2080 elderly patients admitted to the ICU within 24 h after cardiac surgery were included in the study, of whom 419 (20.1%) developed postoperative delirium. Baseline characteristics of the participants are shown in Table 1. Their median MFI score was 2 (interquartile range [IQR]: 1–3). They were divided into two groups according to their frailty status: frail (*n* = 614) and non‐frail (*n* = 1466). Within the study, the median age for the entire cohort was approximately 74 years (IQR: 66.73–79.64 years), 642 (30.9%) were female, and the majority (76.2%) were Caucasian. The median MFI for this cohort was 2 (IQR: 1–3). In general, the frail group had longer overall and ICU hospital stays, greater disease severity, and were likely to have more comorbidities compared to the non‐frail population. In addition, frail patients were more likely to have worse outcomes. Specifically, the frail group had a significantly higher prevalence of delirium (29.2% vs. 16.4%) and pressure injury (11.1% vs. 4.8%) than the non‐frail group (both *p* < 0.05). We also divided the participants into two groups for comparison: those without delirium and those with delirium (Table S3). Patients with delirium were typically older, more severely ill, required more mechanical ventilation, had longer hospital and ICU stays, and had higher in‐hospital mortality.

**TABLE 1:** Characteristics of the patients included in the study.

Characteristics	Total (*n* = 2080)	Non‐frail group^a^ (*n* = 1466)	Frail group (*n* = 614)	*p*‐value
Personal characteristics				
Age (years old)	74.27 (69.73, 79.64)	74.51 (69.74, 80.11)	73.77 (69.70, 78.76)	0.081
Sex (%)				0.211
Male	1438 (69.1)	1001 (68.3)	437 (71.2)	
Female	642 (30.9)	465 (31.7)	177 (28.8)	
Race (%)				0.021*
White	1585 (76.2)	1138 (77.6)	447 (72.8)	
Other	495 (23.8)	328 (22.4)	167 (27.2)	
Marital status (%)				0.041
Married	1291 (62.1)	931 (63.5)	360 (58.6)	
Unmarried	789 (37.9)	535 (36.5)	254 (41.4)	
Hospital LOS (days)	7.28 (5.53, 10.69)	6.75 (5.26, 9.30)	9.25 (6.72, 13.85)	<0.001*
ICU LOS (days)	2.10 (1.29, 3.37)	2.00 (1.27, 3.25)	2.34 (1.35, 4.36)	<0.001*
MFI	2.00 (1.00, 3.00)	1.00 (1.00, 2.00)	3.00 (3.00, 4.00)	<0.001*
Lifestyle				
Tobacco use (%)				0.802
Yes	509 (24.5)	356 (24.3)	153 (24.9)	
No	1571 (75.5)	1110 (75.7)	461 (75.1)	
Alcohol abuse (%)				0.743
Yes	49 (2.4)	33 (2.3)	16 (2.6)	
No	2031 (97.6)	1433 (97.7)	598 (97.4)	
Scores				
Braden score	13 (12, 14)	13 (12, 14)	13 (12, 14)	0.155
SOFA	6 (4, 8)	5 (4, 7)	7 (5, 9)	<0.001*
APSIII	36 (29, 48)	35 (28, 46)	40 (31, 55)	<0.001*
Vital signs				
Temperature, °C	36.44 (35.90, 36.72)	36.44 (35.84, 36.72)	36.50 (36.10, 36.80)	<0.001*
Heart rate, beats/min	80 (73, 85)	80 (73, 84.75)	80 (74, 87)	0.009*
MAP, mmHg	76 (69, 85)	76 (69, 85)	76 (68, 84)	0.804
Respiration rate, breaths/min	16 (14, 18)	16 (14, 17)	16 (14, 18)	<0.001*
Laboratory findings				
Creatinine, mg/dL	0.9 (0.7, 1.1)	0.8 (0.7, 1.0)	1.0 (0.8, 1.3)	<0.001*
White blood cell counts, 10^9^/L	11.5 (8.7, 15.3)	11.4 (8.6, 15.1)	11.7 (8.9, 15.7)	0.062
Hemoglobin, g/dL	9.2 (8.1, 10.5)	9.3 (8.2, 10.5)	9.1 (7.9, 10.3)	0.028*
Albumin, g/dL	3.9 (3.6, 4.3)	4 (3.6, 4.3)	3.8 (3.4, 4.2)	<0.001*
Comorbidities				
Myocardial infarct (%)				<0.001*
Yes	581 (27.9)	186 (12.7)	395 (64.3)	
No	1499 (72.1)	1280 (87.3)	219 (35.7)	
Congestive heart failure (%)				<0.001*
Yes	569 (27.4)	279 (19.0)	290 (47.2)	
No	1511 (72.6)	1187 (81.0)	324 (52.8)	
Peripheral vascular disease (%)				<0.001*
Yes	337 (16.2)	187 (12.8)	150 (24.4)	
No	1743 (83.8)	1279 (87.2)	464 (75.6)	
Cerebrovascular disease (%)				<0.001*
Yes	236 (11.3)	115 (7.8)	121 (19.7)	
No	1844 (88.7)	1351 (92.2)	493 (80.3)	
Chronic pulmonary disease (%)				<0.001*
Yes	480 (23.1)	285 (19.4)	195 (31.8)	
No	1600 (76.9)	1181 (80.6)	419 (68.2)	
Diabetes (%)				<0.001*
Yes	760 (36.5)	349 (23.8)	411 (66.9)	
No	1320 (63.5)	1117 (76.2)	203 (33.1)	
Hypertension (%)				<0.001*
Yes	1235 (59.4)	914 (62.3)	321 (52.3)	
No	845 (40.6)	552 (37.7)	293 (47.7)	
Renal disease (%)				<0.001*
Yes	433 (20.8)	237 (16.2)	196 (31.9)	
No	1647 (79.2)	1229 (83.8)	418 (68.1)	
Malignant cancer (%)				0.277
Yes	60 (2.9)	38 (2.6)	22 (3.6)	
No	2020 (97.1)	1428 (97.4)	592 (96.4)	
Liver disease (%)				0.099
Yes	66 (3.2)	40 (2.7)	26 (4.2)	
No	2014 (96.8)	1426 (97.3)	588 (95.8)	
Depression (%)				0.22
Yes	203 (9.8)	135 (9.2)	68 (11.1)	
No	1877 (90.2)	1331 (90.8)	546 (88.9)	
Treatment				
Mechanical ventilation (%)				<0.001*
Yes	1352 (65.0)	914 (62.3)	438 (71.3)	
No	728 (35.0)	552 (37.7)	176 (28.7)	
Benzodiazepines (%)				0.001
Yes	371 (17.8)	235 (16.0)	136 (22.1)	
No	1709 (82.2)	1231 (84.0)	478 (77.9)	
Surgery type (%)				<0.001*
CABG	1072 (51.5)	671 (45.8)	401 (65.3)	
Valve surgery	495 (23.8)	419 (28.6)	76 (12.4)	
Aortic replacement	39 (1.9)	30 (2.0)	9 (1.5)	
Combined cardiac surgery	474 (22.8)	346 (23.6)	128 (20.8)	
Outcomes				
Delirium (%)				<0.001*
Yes	419 (20.1)	240 (16.4)	179 (29.2)	
No	1661 (79.9)	1226 (83.6)	435 (70.8)	
Pressure injury (%)				<0.001*
Yes	138 (6.6)	70 (4.8)	68 (11.1)	
No	1942 (93.4)	1396 (95.2)	546 (88.9)	
In‐hospital mortality (%)				0.112
Alive	2059 (99.0)	1455 (99.2)	604 (98.4)	
Expired	21 (1.0)	11 (0.8)	10 (1.6)	

Abbreviations: APSIII, Acute Physiology Score III; CABG, coronary artery bypass grafting; ICU, intensive care unit; LOS, length of stay; MAP, mean arterial blood pressure; MFI, modified frailty index; SOFA, sequential organ failure assessment.

^a^ In our study, frailty status was defined based on the total score of the MFI: a score greater than 3 indicated frailty, a score of 1–2 indicated pre‐frailty, and a score of 0 indicated no frailty. For the purpose of this study, a binary classification of pre‐frailty status was performed using the MFI. Patients classified as “pre‐frail” and “non‐frail” were collectively referred to as “non‐frail” patients.

* Significant difference between older patients after cardiac surgery between two groups (*p* < 0.05).

### 3.2 Frailty and adverse outcomes

We used a restricted cubic spline to illustrate the relationship between MFI and POD (Figure S2). There was no significant non‐linear relationship between the OR of POD and MFI. Instead, as MFI increased, the OR for POD showed a consistent upward trend. The results of multivariable logistic regression suggested a significant positive association between MFI and the risk of both delirium (adjusted OR: 1.18, 95% CI: 1.07–1.30, *p* = 0.001) and pressure injury (adjusted OR: 1.39, 95% CI: 1.20–1.60, *p* < 0.001) (Table 2).

**TABLE 2:** Logistic regression: Association between MFI (as a continuous variable) and primary/secondary outcomes.

Outcomes	OR (95% CI)	*p*‐value
Delirium^a^		
Unadjusted	1.31 (1.20–1.42)	<0.001*
Adjusted	1.18 (1.07–1.30)	0.001*
Pressure injury^a^		
Unadjusted	1.52 (1.34–1.72)	<0.001*
Adjusted	1.39 (1.20–1.60)	<0.001*

*Note*: Delirium was adjusted for age, sex, race, marital status, APSIII, SOFA, albumin, creatinine, hemoglobin, white blood cell, surgery type, alcohol abuse, tobacco use, use of benzodiazepines and mechanical ventilation; Pressure injury was adjusted for age, sex, race, marital status, APSIII, SOFA, Braden score and surgery type.

Abbreviations: CI, confidence intervals; OR, odds ratios.

^a^ Logistic regression models were used to calculate odds ratios (OR) with 95% confidence intervals (CI).

* Significant difference between older patients after cardiac surgery between two groups (*p* < 0.05).

For further analysis, patients were categorized according to their frailty score (Table 3). Compared to non‐frail patients, frail patients had a significantly higher risk of delirium (adjusted OR: 1.61, 95% CI: 1.23–2.10, *p* < 0.001, *E*‐value: 1.85) and pressure injuries (adjusted OR: 1.90, 95% CI: 1.26–2.85, *p* = 0.002, *E*‐value: 3.12). These findings underscore that frailty is a significant risk factor for adverse outcomes in older patients undergoing cardiac surgery.

**TABLE 3:** Logistic regression: Association between frailty and primary/secondary outcomes.

	Non‐frail group^a^	Frail group	*p*‐value	*E*‐value (lower limit of the 95% CIs)
		OR (95% CI)		
Delirium^b^				
Unadjusted	Reference	2.10 (1.68, 2.62)	<0.001*	Not applicable
Adjusted	Reference	1.61 (1.23, 2.10)	<0.001*	1.85 (1.46)
Pressure injury^b^				
Unadjusted	Reference	2.48 (1.75, 3.52)	<0.001*	Not applicable
Adjusted	Reference	1.90 (1.26, 2.85)	0.002*	3.21 (1.83)

*Note*: Delirium was adjusted for age, sex, race, marital status, APSIII, SOFA, albumin, creatinine, hemoglobin, white blood cell, surgery type, alcohol abuse, tobacco use, cerebrovascular disease, use of benzodiazepines and mechanical ventilation; Pressure injury was adjusted for age, sex, race, marital status, APSIII, SOFA, Braden score and surgery type.

Abbreviations: CI, confidence intervals; OR, odds ratios.

^a^ In our study, frailty status was defined based on the total score of the MFI: a score greater than 3 indicated frailty, a score of 1–2 indicated pre‐frailty, and a score of 0 indicated no frailty. For the purpose of this study, a binary classification of pre‐frailty status was performed using the MFI. Patients classified as “pre‐frail” and “non‐frail” were collectively referred to as “non‐frail” patients.

^b^ Logistic regression models were used to calculate odds ratios (OR) with 95% confidence intervals (CI).

* Significant difference between older patients after cardiac surgery between two groups (*p* < 0.05).

### 3.3 Subgroup analysis

**FIGURE 2:**
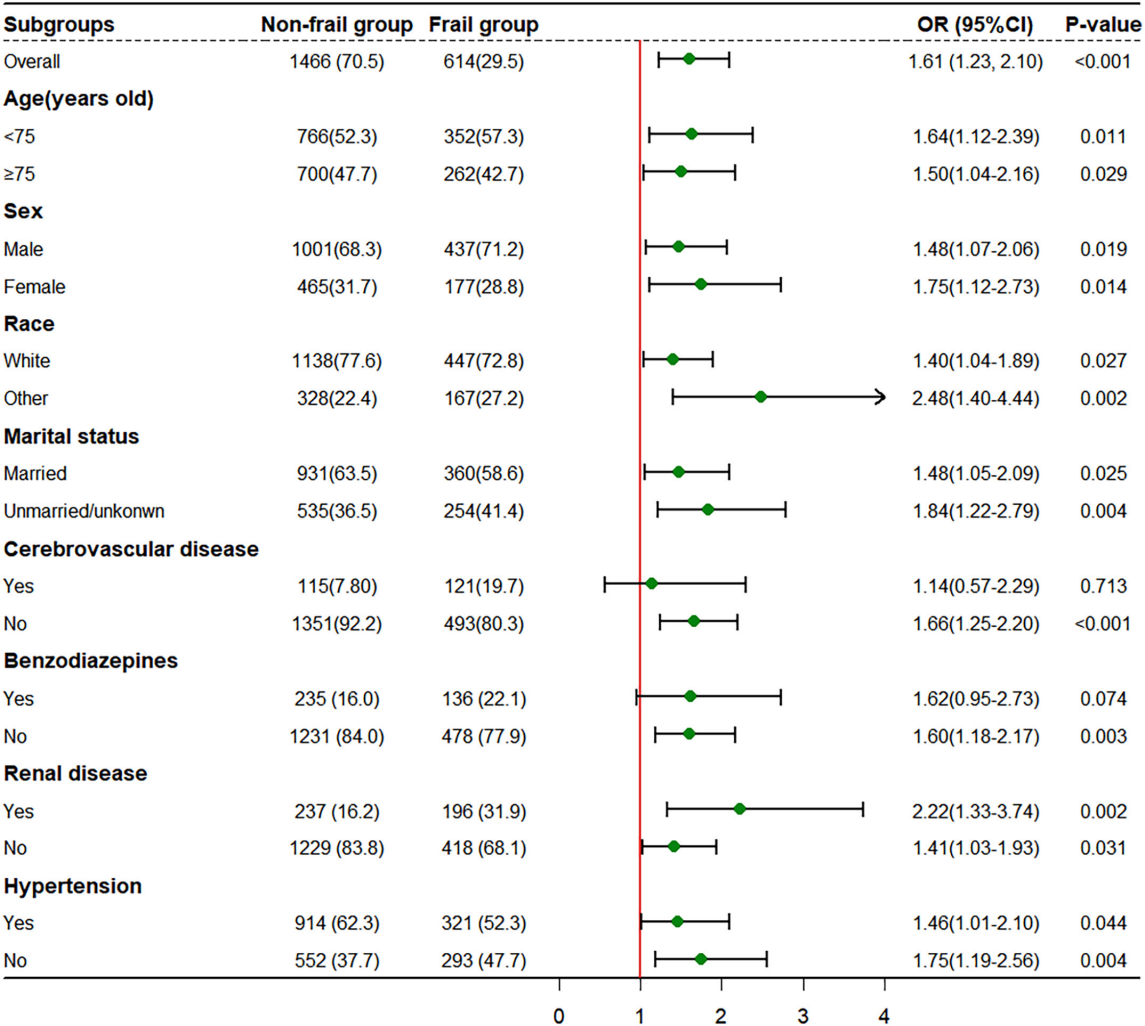
Forest plot for subgroup analysis. CI, confidence interval; OR, odds ratio. Frailty status was defined based on the total score of the MFI: a score greater than 3 indicated frailty, a score of 1–2 indicated pre‐frailty, and a score of 0 indicated no frailty. For the purpose of this study, a binary classification of pre‐frailty status was performed using the MFI. Patients classified as “pre‐frail” and “non‐frail” were collectively referred to as “non‐frail” patients.

We performed a subgroup analysis to examine the association between frailty and the primary outcome of delirium in different subpopulations (Figure 2). The results of this study indicate that the association between frailty and delirium is not statistically significant in the subgroups of cerebrovascular disease and the use of benzodiazepine. However, we observed statistically significant associations in other subgroups.

### 3.4 Sensitivity analysis

Sensitivity analyses were performed to further explore the association between frailty and delirium. Using the PSM method, we achieved balance between groups and better comparability between covariates. The relevant changes in SMD are shown in Figure S3. Performing univariable and multivariable logistic regression on the matched population, we obtained consistent results with the original population (Table S4), namely that frailty and older postcardiac surgery patients were associated with the risk of delirium.

To further verify the robustness of the study results and to exclude potential confounders, we performed adjustment analyses with two different models. In Model 1, we focused specifically on patients who survived hospitalization, a total of 2059 individuals. Model 2 was limited to patients who underwent combined cardiac surgery, with a sample size of 474, while Model 3 included only patients diagnosed with sepsis, with a sample size of 1109. Frailty was identified as an independent risk factor for delirium in these models, and the results were consistently robust (Table S5). A statistically significant association between frailty and delirium was observed in a sensitivity analysis using the full data set after excluding patients with missing data (Table S6). Furthermore, the same pattern was found when different combinations of covariates were analyzed (Table S7).

In addition, we calculated the *E*‐value, a measure of the effect that unaccounted for confounders may have on the study results. In our study examining the association between frailty and POD, we calculated an adjusted OR of 1.61 (95% CI: 1.23–2.10). Sensitivity analysis of this study yielded an *E*‐value of 1.85 with a lower limit of the 95% CI of 1.46. This suggests that there may be unmeasured confounders that could account for an association between frailty and POD, as a confounding variable associated with both exposure and outcome by an odds ratio of at least 1.85 could explain the observed association.

## 4 DISCUSSION

Given the potentially detrimental effects of POD in elderly patients after cardiac surgery, it is imperative to accurately assess the risk of delirium in these individuals. In this study, using a large, publicly available database, we conducted an in‐depth analysis of elderly patients admitted to the ICU within 24 h of cardiac surgery. The results showed that frailty (as indicated by the MFI) remains an independent predictor of PDO, even after controlling for other potential confounders. Compared to non‐frail patients, those with frailty may be at significantly increased risk of PDO. In addition, a similar pattern was observed when stress injuries were considered. These findings underscore the utility of the MFI as a tool to screen for frailty in elderly cardiac surgery patients in the ICU and highlight the adverse consequences of frailty in this patient population.

With the growth of the world's elderly population, frailty has gradually become a focus of international geriatric medicine and public health.^9^ While it is widely accepted that health challenges increase with age, what is more compelling is that frailty may predict morbidity and mortality after surgery in the elderly more accurately than chronological age.^41^, ^42^ A recent systematic review and meta‐analysis of cardiac surgery patients reported that frailty was identified as a key predictor associated with increased short‐ and long‐term mortality, increased postoperative complications, increased rates of transition to nursing facilities, increased risk of readmission, and decreased health‐related quality of life after surgery.^43^ Previous studies have examined the association between frailty and delirium in patients after cardiac surgery and found an increased risk of POD due to frailty.^16^, ^17^, ^44^ However, these studies were limited by sample size. Our large study of 2080 elderly patients after cardiac surgery further confirmed this association. Although the exact mechanism behind this association remains elusive, the current evidence provides us with a number of plausible explanations. Geriatricians and cardiovascular researchers describe frailty as a multidimensional syndrome that leads to physiological and psychological vulnerabilities, particularly in high‐risk settings such as the ICU.^45^ In addition, neuroinflammation has been implicated as a primary cause of delirium, and systemic inflammation associated with cardiac surgery may be exacerbated in frail patients.^46^ Coupled with the fact that cardiac surgery is a significant stressor associated with frailty and chronic systemic inflammation, this adds to the complexity of the inflammatory response during such surgery and consequently increases the risk of POD.^47^ Notably, individuals with frailty often exhibit cognitive decline, and impaired cognition is a significant risk factor for POD and other adverse surgical outcomes.^48^ Finally, the lack of social support and feelings of loneliness in frail patients may also increase their risk of delirium.^49^ Therefore, comprehensive perioperative care for this vulnerable group, such as adequate nutritional support, proper medication management, psychological support, and early rehabilitation, may help reduce their risk of delirium.

Pressure injuries (commonly known as pressure ulcers) are a common complication after cardiac surgery and represent a significant burden for both patients and the healthcare system.^50^ Despite the importance of this issue, current clinical trials rarely focus specifically on cardiac surgery patients.^50^ In this study, we focused on elderly patients after cardiac surgery and found that frailty is a significant risk factor for the development of pressure injuries. This is consistent with the research by Lv and colleagues on geriatric patients with multiple comorbidities in five tertiary hospitals in Sichuan Province.^51^ Frail patients are more susceptible to pressure injuries due to reduced physiological reserves, fragile skin and limited mobility.^52^ In addition, potential malnutrition and chronic inflammation increase the risk.^47^ Therefore, early skin assessment and preventive measures are critical for elderly patients following cardiac surgery, especially those at high risk due to frailty. This will help reduce the incidence of pressure injuries, improve patient quality of life and reduce the burden on the healthcare system.

For patients undergoing cardiac surgery, accurate assessment of frailty status in the busy hospital environment is critical for predicting outcomes and developing appropriate management strategies. Based on the available data, we chose the validated MFI as our assessment tool. However, frailty is a multidimensional concept, encompassing both biomedical factors and social‐psychological dimensions, adding complexity to the search for an appropriate assessment tool.^53^ While the MFI provides a structured method of assessment, a more comprehensive and in‐depth approach remains to be explored. To better understand frailty, it is imperative to conduct holistic assessments from physical, nutritional, cognitive, and social‐psychological perspectives.^54^ Thanks to modern technology, particularly advances in artificial intelligence and wearable devices, we can now accurately capture patient needs and frailty data, facilitating effective identification of high‐risk individuals.^55^

In the management and prevention of frailty, multimodal therapeutic strategies such as improving appetite, reducing the inflammatory response, caloric supplementation, and exercise to improve physical capacity and quality of life are invaluable.^56^ However, while we recognize the importance of these interventions, allocating resources and implementing effective interventions in real‐world medical practice remains a challenge. To ensure that research findings truly benefit clinical practice, these issues warrant further investigation. In conclusion, the assessment and management of frailty in elderly patients at the time of cardiac surgery is an important but complex task. It requires close collaboration between multidisciplinary teams and technological support to provide patients with the most scientifically sound and holistic treatment plans.^57^

This study has several strengths. First and foremost, our study has a larger sample size compared to previous research, which lends greater credibility to our findings. Second, the assessment of frailty and delirium in the ICU setting is challenging. However, by using validated novel approaches to assess frailty (using the MFI) and delirium (using the CAM‐ICU and review of nursing notes), we have further minimized the risk of misclassification. This is particularly important as the diagnosis of frailty and delirium is often complicated in clinical scenarios. In addition, to ensure the robustness of our results, we performed several sensitivity analyses, in particular by introducing the *E*‐value, which helps to interpret the influence of potential unmeasured confounders.

However, our study has limitations. The main limitation is the single‐center retrospective design, which may limit the broad applicability of the study results. In addition, we only examined the association between frailty and POD without establishing a definitive causal relationship. Due to database limitations, we lacked certain key variables that could influence outcomes, such as patients' cardiac function classification, cognitive function, ability to self‐care for activities of daily living, and duration of extracorporeal circulation during surgery. There is also a slight imprecision in the MFI score, especially considering the coexistence of ICD‐9 and ICD‐10 codes in the MIMIC‐IV database and our use of their conversion to construct the MFI. Similarly, the use of ICD code R26 to assess patients' functional status may have introduced certain inaccuracies in the MFI. Finally, our study did not include information on the type, duration, and severity of delirium, which may limit our understanding of the relationship between frailty and delirium. Future studies should delve deeper into the specific causal relationship between frailty and delirium, and consider including more surgery‐related variables and detailed delirium information. At the same time, multicenter prospective studies and larger sample sizes will improve the generalizability of study results and potentially help to better understand and predict the association between frailty and delirium in elderly patients after cardiac surgery.

## 5 CONCLUSION

In conclusion, our results indicate a positive association between MFI and the risk of POD and pressure injuries in elderly patients after cardiac surgery. Compared with non‐frail patients, frail patients may be at higher risk for POD and pressure injuries. This provides valuable insights for healthcare professionals and highlights the importance of accurate assessment and effective management of frailty status in older patients undergoing cardiac surgery.

## AUTHOR CONTRIBUTIONS

All authors made a significant contribution to the work reported either in the conception, study design, execution, acquisition of data, analysis and interpretation or in all these areas; took part in drafting, revising, or critically reviewing the article; gave final approval for the version to be published; have agreed on the journal to which the article has been submitted; and agree to be accountable for all aspects of the work.

## FUNDING INFORMATION

The work was supported by the Guangdong Provincial Key Laboratory of Traditional Chinese Medicine Informatization (2021B1212040007), the Clinical Frontier Technology Program of the First Affiliated Hospital of Jinan University, China (No. JNU1AF‐CFTP‐2022‐a01235), and the Science and Technology Projects in Guangzhou, China (No. 202201020054, No. 2023A03J1032).

## CONFLICT OF INTEREST STATEMENT

The authors declare no conflicts of interest.

## INFORMED CONSENT

Data extracted from the MIMIC‐IV database do not require individual informed consent because the research data are publicly available, and all patient data are de‐identified.

## Supporting information


Data S1

## Data Availability

The data were available on the MIMIC‐IV website at https://mimic.physionet.org/. The data in this article can be reasonably applied to the corresponding authors.
