# Supplementary material for: Association between modified frailty index and postoperative delirium in patients after cardiac surgery: A cohort study of 2080 older adults
Source: CNS Neurosci Ther. 2024 Jun 25;30(6):e14762. doi: 10.1111/cns.14762 (PMC11199331; doi:10.1111/cns.14762)
Supplement: Supplementary file 1 — Data S1 [file CNS-30-e14762-s001.docx]

**Legends for Supplementary Tables and Figures**

**Supplementary Table 1** ICD-9 and ICD-10 codes for identifying cardiac surgeries

**Supplementary Table 2** ICD-9 and ICD-10 codes used in modified frailty index construction

**Supplementary Table 3** Characteristics of the patients included in the study

**Supplementary Table 4** Logistic regression after PSM: association between frailty and postoperative delirium.

**Supplementary Table 5** Sensitivity analysis: Association between frailty and postoperative delirium in special populations.

**Supplementary Table 6** Association between frailty and primary/secondary outcomes analysis using the full dataset (n=1952).

**Supplementary Table 7** Association between frailty and delirium sensitive analysis using different combinations of covariates.

**Supplementary Figure 1** The missing rate of study variables.

**Supplementary Figure 2** Restricted cubic spline regression was used to analyze the potential association between modified frailty index scores and risk of delirium in older patients after cardiac surgery.

**Supplementary Figure 3** Standardized mean differences before and after propensity score matching.

**Supplementary Table 1** ICD-9 and ICD-10 codes for identifying cardiac surgeries

| ICD codes | ICD version | Long title |
| --- | --- | --- |
| 3511 | 9 | Open heart valvuloplasty of aortic valve without replacement |
| 3512 | 9 | Open heart valvuloplasty of mitral valve without replacement |
| 3514 | 9 | Open heart valvuloplasty of tricuspid valve without replacement |
| 3521 | 9 | Open and other replacement of aortic valve with tissue graft |
| 3522 | 9 | Open and other replacement of aortic valve |
| 3523 | 9 | Open and other replacement of mitral valve with tissue graft |
| 3524 | 9 | Open and other replacement of mitral valve |
| 3527 | 9 | Open and other replacement of tricuspid valve with tissue graft |
| 3611 | 9 | (Aorto)coronary bypass of one coronary artery |
| 3612 | 9 | (Aorto)coronary bypass of two coronary arteries |
| 3613 | 9 | (Aorto)coronary bypass of three coronary arteries |
| 3614 | 9 | (Aorto)coronary bypass of four or more coronary arteries |
| 3615 | 9 | Single internal mammary-coronary artery bypass |
| 3616 | 9 | Double internal mammary-coronary artery bypass |
| 3619 | 9 | Other bypass anastomosis for heart revascularization |
| 3845 | 9 | Resection of vessel with replacement, thoracic vessels |
| 0210089 | 10 | Bypass Coronary Artery, One Artery from Left Internal Mammary with Zooplastic Tissue, Open Approach |
| 0210093 | 10 | Bypass Coronary Artery, One Artery from Coronary Artery with Autologous Venous Tissue, Open Approach |
| 0210099 | 10 | Bypass Coronary Artery, One Artery from Left Internal Mammary with Autologous Venous Tissue, Open Approach |
| 021009W | 10 | Bypass Coronary Artery, One Artery from Aorta with Autologous Venous Tissue, Open Approach |
| 02100A3 | 10 | Bypass Coronary Artery, One Artery from Coronary Artery with Autologous Arterial Tissue, Open Approach |
| 02100A8 | 10 | Bypass Coronary Artery, One Artery from Right Internal Mammary with Autologous Arterial Tissue, Open Approach |
| 02100A9 | 10 | Bypass Coronary Artery, One Artery from Left Internal Mammary with Autologous Arterial Tissue, Open Approach |
| 02100AW | 10 | Bypass Coronary Artery, One Artery from Aorta with Autologous Arterial Tissue, Open Approach |
| 02100Z3 | 10 | Bypass Coronary Artery, One Artery from Coronary Artery, Open Approach |
| 02100Z8 | 10 | Bypass Coronary Artery, One Artery from Right Internal Mammary, Open Approach |
| 02100Z9 | 10 | Bypass Coronary Artery, One Artery from Left Internal Mammary, Open Approach |
| 0211093 | 10 | Bypass Coronary Artery, Two Arteries from Coronary Artery with Autologous Venous Tissue, Open Approach |
| 0211099 | 10 | Bypass Coronary Artery, Two Arteries from Left Internal Mammary with Autologous Venous Tissue, Open Approach |
| 021109W | 10 | Bypass Coronary Artery, Two Arteries from Aorta with Autologous Venous Tissue, Open Approach |
| 02110A9 | 10 | Bypass Coronary Artery, Two Arteries from Left Internal Mammary with Autologous Arterial Tissue, Open Approach |
| 02110AW | 10 | Bypass Coronary Artery, Two Arteries from Aorta with Autologous Arterial Tissue, Open Approach |
| 02110Z3 | 10 | Bypass Coronary Artery, Two Arteries from Coronary Artery, Open Approach |
| 02110Z8 | 10 | Bypass Coronary Artery, Two Arteries from Right Internal Mammary, Open Approach |
| 02110Z9 | 10 | Bypass Coronary Artery, Two Arteries from Left Internal Mammary, Open Approach |
| 0212093 | 10 | Bypass Coronary Artery, Three Arteries from Coronary Artery with Autologous Venous Tissue, Open Approach |
| 0212099 | 10 | Bypass Coronary Artery, Three Arteries from Left Internal Mammary with Autologous Venous Tissue, Open Approach |
| 021209W | 10 | Bypass Coronary Artery, Three Arteries from Aorta with Autologous Venous Tissue, Open Approach |
| 02120AW | 10 | Bypass Coronary Artery, Three Arteries from Aorta with Autologous Arterial Tissue, Open Approach |
| 02120Z8 | 10 | Bypass Coronary Artery, Three Arteries from Right Internal Mammary, Open Approach |
| 02120Z9 | 10 | Bypass Coronary Artery, Three Arteries from Left Internal Mammary, Open Approach |
| 0213093 | 10 | Bypass Coronary Artery, Four or More Arteries from Coronary Artery with Autologous Venous Tissue, Open Approach |
| 0213099 | 10 | Bypass Coronary Artery, Four or More Arteries from Left Internal Mammary with Autologous Venous Tissue, Open Approach |
| 021309W | 10 | Bypass Coronary Artery, Four or More Arteries from Aorta with Autologous Venous Tissue, Open Approach |
| 02130A3 | 10 | Bypass Coronary Artery, Four or More Arteries from Coronary Artery with Autologous Arterial Tissue, Open Approach |
| 02130Z8 | 10 | Bypass Coronary Artery, Four or More Arteries from Right Internal Mammary, Open Approach |
| 02QF0ZZ | 10 | Repair Aortic Valve, Open Approach |
| 02QG0ZZ | 10 | Repair Mitral Valve, Open Approach |
| 02RF08Z | 10 | Replacement of Aortic Valve with Zooplastic Tissue, Open Approach |
| 02RF0JZ | 10 | Replacement of Aortic Valve with Synthetic Substitute, Open Approach |
| 02RF0KZ | 10 | Replacement of Aortic Valve with Nonautologous Tissue Substitute, Open Approach |
| 02RG08Z | 10 | Replacement of Mitral Valve with Zooplastic Tissue, Open Approach |
| 02RG0JZ | 10 | Replacement of Mitral Valve with Synthetic Substitute, Open Approach |
| 02RG0KZ | 10 | Replacement of Mitral Valve with Nonautologous Tissue Substitute, Open Approach |
| 02RJ08Z | 10 | Replacement of Tricuspid Valve with Zooplastic Tissue, Open Approach |
| 02RW0JZ | 10 | Replacement of Thoracic Aorta, Descending with Synthetic Substitute, Open Approach |
| 02RX08Z | 10 | Replacement of Thoracic Aorta, Ascending/Arch with Zooplastic Tissue, Open Approach |
| 02RX0JZ | 10 | Replacement of Thoracic Aorta, Ascending/Arch with Synthetic Substitute, Open Approach |

**Supplementary Table 2** ICD-9 and ICD-10 codes used in modified frailty index construction

| **No.** | **Items** | **ICD-10 Description** | **ICD-10 Code** | **ICD-9 Code** |
| --- | --- | --- | --- | --- |
| **1** | History of Diabetes Mellitus | Diabetes mellitus due to underlying condition; Drug or chemical induced diabetes mellitus; Type 1 diabetes mellitus; Type 2 diabetes mellitus; Other specified diabetes mellitus; Encounter for screening for diabetes mellitus. | E08* OR E09* OR E10* OR E11* OR E13* OR Z131* | 249* OR 250* OR 3572 OR V771 |
| **2** | History of Congestive Heart Failure | Heart failure, unspecified. | I50.9 | 4280 |
| **3** | History of Hypertension Requiring Medication | Hypertensive disease. | I1* | 40* |
| **4** | History of either Transient Ischemic Attack or Cerebrovascular Accident | Transient cerebral ischemic attacks and related syndromes; Nontraumatic subarachnoid hemorrhage; Nontraumatic intracerebral hemorrhage; Other and unspecified nontraumatic intracranial hemorrhage; Cerebral infarction; Personal history of transient ischemic attack (TIA), and cerebral infarction without residual deficits. | G45* OR I60* OR I61* OR I62* OR I63* OR Z86.73 | 430 OR 431 OR 432* OR 43301 OR 43311 OR 43321 OR 43331 OR 43381 OR 43391 OR 43401 OR 43411 OR 43491 OR 435* OR V1254 |
| **5** | Functional Status (Loss Function) | Abnormalities of gait and mobility. | R26* | 7197 OR 7812 |
| **6** | History of Myocardial Infarction | ST elevation (STEMI) and non-ST elevation (NSTEMI) myocardial infarction; Subsequent ST elevation (STEMI) and non-ST elevation (NSTEMI) myocardial infarction; Certain current complications following ST elevation (STEMI) and non-ST elevation (NSTEMI) myocardial infarction (within the 28 days period); Old myocardial infarction; Cardiac septal defect, acquired. | I21* OR I22* OR I23* OR I252 OR I510 | 410* OR 4110 OR 412 OR 4297* |
| **7** | History of either Peripheral Vascular Disease or Rest Pain | Atherosclerosis of renal artery; Atherosclerosis of native arteries of the extremities; Atherosclerosis of other arteries; Other and unspecified atherosclerosis; Other aneurysm; Other peripheral vascular diseases; Embolism and thrombosis of arteries of the upper extremities; Embolism and thrombosis of arteries of the lower extremities; Embolism and thrombosis of arteries of extremities, unspecified; Embolism and thrombosis of iliac artery; Embolism and thrombosis of other arteries; Embolism and thrombosis of unspecified artery; Atheroembolism; Phlebitis and thrombophlebitis; Portal vein thrombosis; Other venous embolism and thrombosis; Varicose veins of lower extremities; Esophageal varices; Varicose veins of other sites; Personal history of thrombophlebitis. | I70.1 OR I70.2* OR I70.8 OR I70.9* OR I72* OR I73* OR I74.2 OR I74.3 OR I74.4 OR I74.5 OR I74.8 OR I74.9 OR I75* OR I80* OR I81* OR I82* OR I83* OR I85* OR I86* OR Z8672 | 4401 OR 4402* OR 4404 OR 4408 OR 4409 OR 442* OR 443* OR 4442* OR 4448* OR 4449 OR 445* OR 451* OR 452* OR 453* OR 454* OR 456* OR V1252 |
| **8** | History of Cerebrovascular Accident with Neurological Deficit | Sequelae of cerebrovascular disease. | I69* | 438* |
| **9** | History of either COPD or Pneumonia | Viral pneumonia, not elsewhere classified; Pneumonia due to Streptococcus pneumoniae; Pneumonia due to Hemophilus influenzae; Bacterial pneumonia, not elsewhere classified; Pneumonia due to other infectious organisms, not elsewhere classified; Pneumonia in diseases classified elsewhere; Pneumonia, unspecified organism; Emphysema; Other chronic obstructive pulmonary disease; Personal history of pneumonia (recurrent). | J12* OR J13* OR J14* OR J15* OR J16* OR J17* OR J18* OR J43* OR J44* OR Z78.01 | 480* OR 481* OR 482* OR 483* OR 484* OR 485* OR 486* OR 491* OR 492* OR V1261 |
| **10** | History of either prior PCI, CABG, or angina | Angina pectoris; Atherosclerotic heart disease of native coronary artery with angina pectoris; Atherosclerosis of coronary artery bypass graft(s), unspecified, with angina pectoris; Atherosclerosis of autologous vein coronary artery bypass graft(s) with angina pectoris; Atherosclerosis of autologous artery coronary artery bypass graft(s) with angina pectoris; Atherosclerosis of non-autologous biological coronary artery bypass graft(s) with angina pectoris; Atherosclerosis of other coronary artery bypass graft(s) with angina pectoris; Presence of aortocoronary bypass graft; Presence of coronary angioplasty implant and graft; Coronary angioplasty status. | I20* OR I2511 OR I2570 OR I2571 OR I2572 OR I2573 OR I2579 OR Z951 OR Z955 OR Z9861 | 4111 OR 413* OR 41402 OR 41403 OR 41404 OR 41405 OR V4581 OR V4582 OR V472 |
| **11** | History of Impaired Sensorium | Disturbances of skin sensation; Disturbances of smell and taste; Visual disturbances; Blindness and low vision; Conductive and sensorineural hearing loss; Other and unspecified hearing loss; Dual sensory impairment. | R20* OR R43* OR H53* OR H54* OR H90* OR H91* OR Z73.82 | 7820 OR 7811 OR V415 OR 368* OR 369* OR V410 OR 389* OR 3881* OR 3882 OR V412 OR V4985 OR V484 OR V485 OR V493 |

Note:

"*" means all subcodes contained in this code.

"AND", "OR" and "NOT" follow the logic of Boolean operations.

**Supplementary Table 3** Characteristics of the patients included in the study.

| Variable | Total (n=2080) | Non-delirium (n=1661) | Delirium (n=419) | P-value |
| --- | --- | --- | --- | --- |
| Age (years old) | 74.27 (69.73, 79.64) | 73.56 (69.14, 79.14) | 76.72 (72.20, 81.45) | <0.001* |
| Sex (%) |  |  |  | 0.001* |
| Male | 1438 (69.1) | 1178 (70.9) | 260 (62.1) |  |
| Female | 642 (30.9) | 483 (29.1) | 159 (37.9) |  |
| Race (%) |  |  |  | 0.721 |
| White | 1585 (76.2) | 1269 (76.4) | 316 (75.4) |  |
| Other | 495 (23.8) | 392 (23.6) | 103 (24.6) |  |
| Marital status (%) |  |  |  | 0.036* |
| Married | 1291 (62.1) | 1050 (63.2) | 241 (57.5) |  |
| Unmarried | 789 (37.9) | 611 (36.8) | 178 (42.5) |  |
| Hospital LOS (days) | 7.28 (5.53, 10.69) | 6.95 (5.30, 9.85) | 9.80 (6.91, 16.65) | <0.001* |
| ICU LOS (days) | 2.10 (1.29, 3.37) | 1.57 (1.25, 3.07) | 4.25 (2.31, 8.23) | <0.001* |
| MFI | 2 (1, 3) | 2 (1, 3) | 2 (1, 3) | <0.001* |
| **Lifestyle** |  |  |  |  |
| Tobacco use (%) |  |  |  | 0.448 |
| Yes | 509 (24.5) | 400 (24.1) | 109 (26.0) |  |
| No | 1571 (75.5) | 1261 (75.9) | 310 (74.0) |  |
| Alcohol abuse (%) |  |  |  | 0.042* |
| Yes | 49 (2.4) | 33 (2.0) | 16 (3.8) |  |
| No | 2031 (97.6) | 1628 (98.0) | 403 (96.2) |  |
| **Scores** |  |  |  |  |
| Braden score | 13 (12, 14) | 13 (12, 14) | 13 (12, 14) | 0.034* |
| SOFA | 6 (4, 8) | 5 (4, 7) | 8 (5, 10) | <0.001* |
| APSIII | 36 (29, 48) | 35 (28, 45) | 44 (32, 65) | <0.001* |
| **Vital signs** |  |  |  |  |
| Temperature,℃ | 36.44 (35.90, 36.72) | 36.44 (35.90, 36.72) | 36.50 (35.95, 36.83) | 0.001* |
| Heart rate, beats/min | 80 (73, 85) | 80 (73, 85) | 80 (73, 88) | 0.127 |
| MBP, mmHg | 76 (69, 85) | 76 (69., 84) | 76 (67, 86) | 0.988 |
| Respiration rate, breaths/min | 16 (14, 18) | 16 (14, 17) | 16 (14, 18) | 0.009* |
| **Laboratory parameters** |  |  |  |  |
| Creatinine, mg/L | 0.9 (0.7, 1.1) | 0.9 (0.7, 1.1) | 0.9 (0.7, 1.2) | <0.001* |
| White blood cells counts,10^9^/L | 11.5 (8.7, 15.3) | 11.4 (8.6, 15.0) | 11.8 (8.8, 16.65) | 0.027* |
| Hemoglobin, g/dL | 9.2 (8.1, 10.5) | 9.3 (8.2, 10.6) | 8.8 (7.65, 10.1) | <0.001* |
| Albumin, g/L | 3.9 (3.6, 4.3) | 4.0 (3.6, 4.3) | 3.8 (3.3, 4.2) | <0.001* |
| **Comorbidities** |  |  |  |  |
| Myocardial infarct (%) |  |  |  | 0.129 |
| Yes | 581 (27.9) | 451 (27.2) | 130 (31.0) |  |
| No | 1499 (72.1) | 1210 (72.8) | 289 (69.0) |  |
| Congestive heart failure (%) |  |  |  | <0.001* |
| Yes | 569 (27.4) | 410 (24.7) | 159 (37.9) |  |
| No | 1511 (72.6) | 1251 (75.3) | 260 (62.1) |  |
| Peripheral vascular disease (%) |  |  |  | <0.001* |
| Yes | 337 (16.2) | 228 (13.7) | 109 (26.0) |  |
| No | 1743 (83.8) | 1433 (86.3) | 310 (74.0) |  |
| Cerebrovascular disease (%) |  |  |  | <0.001* |
| Yes | 236 (11.3) | 165 (9.9) | 71 (16.9) |  |
| No | 1844 (88.7) | 1496 (90.1) | 348 (83.1) |  |
| Chronic pulmonary disease (%) |  |  |  | 0.021* |
| Yes | 480 (23.1) | 365 (22.0) | 115 (27.4) |  |
| No | 1600 (76.9) | 1296 (78.0) | 304 (72.6) |  |
| Diabetes (%) |  |  |  | <0.001* |
| Yes | 760 (36.5) | 569 (34.3) | 191 (45.6) |  |
| No | 1320 (63.5) | 1092 (65.7) | 228 (54.4) |  |
| Hypertension (%) |  |  |  | 0.003* |
| Yes | 1235 (59.4) | 1013 (61.0) | 222 (53.0) |  |
| No | 845 (40.6) | 648 (39.0) | 197 (47.0) |  |
| Renal disease (%) |  |  |  | <0.001* |
| Yes | 433 (20.8) | 315 (19.0) | 118 (28.2) |  |
| No | 1647 (79.2) | 1346 (81.0) | 301 (71.8) |  |
| Malignant cancer (%) |  |  |  | 0.398 |
| Yes | 60 (2.9) | 51 (3.1) | 9 (2.1) |  |
| No | 2020 (97.1) | 1610 (96.9) | 410(97.9) |  |
| Liver disease (%) |  |  |  | 0.19 |
| Yes | 66 (3.2) | 48 (2.9) | 18 (4.3) |  |
| No | 2014 (96.8) | 1613 (97.1) | 401 (95.7) |  |
| Depression (%) |  |  |  | 0.02* |
| Yes | 203 (9.8) | 149 (9.0) | 54 (12.9) |  |
| No | 1877 (90.2) | 1512 (91.0) | 365 (87.1) |  |
| **Treatments and drugs** |  |  |  |  |
| Mechanical ventilation (%) |  |  |  | <0.001* |
| Yes | 1352 (65.0) | 1004 (60.4) | 348 (83.1) |  |
| No | 728 (35.0) | 657 (39.6) | 71 (16.9) |  |
| Benzodiazepines (%) |  |  |  |  |
| Yes | 371 (17.8) | 232 (14.0) | 139 (33.2) | <0.001* |
| No | 1709 (82.2) | 1429 (86.0) | 280 (66.8) |  |
| Surgery type (%) |  |  |  | <0.001* |
| CABG | 1072 (51.5) | 904 (54.4) | 168 (40.1) |  |
| Valve surgery | 495 (23.8) | 405 (24.4) | 90 (21.5) |  |
| Aortic replacement | 39 (1.9) | 17 (1.0) | 22 (5.3) |  |
| Combined cardiac surgery | 474 (22.8) | 335 (20.2) | 139 (33.2) |  |
| **Outcomes** |  |  |  |  |
| Delirium (%) |  |  |  | <0.001* |
| Yes | 419 (20.1) | 0 (0.0) | 419 (100.0) |  |
| No | 1661 (79.9) | 1661 (100.0) | 0 (0.0) |  |
| Pressure injury (%) |  |  |  | <0.001* |
| Yes | 138 (6.6) | 64 (3.9) | 74 (17.7) |  |
| No | 1942 (93.4) | 1597 (96.1) | 345 (82.3) |  |
| Hospital expire flag (%) |  |  |  | <0.001* |
| Yes | 21 (1.0) | 8 (0.5) | 13 (3.1) |  |
| No | 2059 (99.0) | 1653 (99.5) | 406 (96.9) |  |

Abbreviations: LOS, length of stay; MFI, modified frailty index; ICU, intensive care unit; MAP, mean arterial blood pressure; SOFA, Sequential Organ Failure Assessment; APSIII: Acute Physiology Score III; CABG, coronary artery bypass grafting.

*Significant difference between older patients after cardiac surgery between two groups (p < 0.05).

**Supplementary Table 4** Logistic regression after PSM: association between frailty and postoperative delirium.

|  | Non-frail group^#^ | Frail group | P-value |
| --- | --- | --- | --- |
|  |  | OR (95% CI) |  |
| Delirium^●^  PSM model  Adjusted PSM model | Reference  Reference | 1.62 (1.25, 2.12)  1.62 (1.20, 2.19) | <0.001*  0.001* |

Abbreviations: OR: odds ratios; CI: confidence intervals; PSM: propensity score matching.

Note:

^#^In our study, frailty status was defined based on the total score of the Modified Frailty Index (MFI): a score greater than 3 indicated frailty, a score of 1-2 indicated pre-frailty, and a score of 0 indicated no frailty. For the purpose of this study, a binary classification of pre-frailty status was performed using the MFI. Patients classified as "pre-frail" and "non-frail" were collectively referred to as "non-frail" patients.

^●^Logistic regression models were used to calculate odds ratios (OR) with 95% confidence intervals (CI).

*Significant difference between older patients after cardiac surgery between two groups (p < 0.05).

Adjusted PSM model for age, sex, race, marital status, Acute Physiology Score III (APSIII), Sequential Organ Failure Assessment (SOFA), albumin, creatinine, hemoglobin, white blood cell, surgery type, alcohol abuse, tobacco use, cerebrovascular disease, use of benzodiazepines and mechanical ventilation.

**Supplementary Table 5** Sensitivity analysis: Association between frailty and postoperative delirium in special populations.

|  | Non-frail group^#^ | Frail group | P-value |
| --- | --- | --- | --- |
|  |  | OR (95% CI) |  |
| Delirium●  Model 1^$^  Unadjusted  Adjusted | Reference  Reference | 2.08 (1.66, 2.60)  1.63 (1.25, 2.14) | <0.001*  <0.001* |
| Delirium●  Model 2^$^  Unadjusted  Adjusted | Reference  Reference | 2.84 (1.85, 4.37)  2.07 (1.25, 3.41) | <0.001*  0.004* |
| Delirium●  Model 3^$^  Unadjusted  Adjusted | Reference  Reference | 3.35 (2.50, 4.50)  2.09 (1.47, 2.96) | <0.001*  <0.001* |

Abbreviations: OR: odds ratios; CI: confidence intervals.

Note:

^$^Model 1 included only in-hospital survivors (n=2059), Model 2 included only patients who underwent combined cardiac surgery (n=474) and Model 2 included only patients with sepsis (n=1109).

^#^In our study, frailty status was defined based on the total score of the Modified Frailty Index (MFI): a score greater than 3 indicated frailty, a score of 1-2 indicated pre-frailty, and a score of 0 indicated no frailty. For the purpose of this study, a binary classification of pre-frailty status was performed using the MFI. Patients classified as "pre-frail" and "non-frail" were collectively referred to as "non-frail" patients.

^●^Logistic regression models were used to calculate odds ratios (OR) with 95% confidence intervals (CI).

*Significant difference between older patients after cardiac surgery between two groups (p < 0.05).

Delirium was adjusted for age, sex, race, marital status, Acute Physiology Score III (APSIII), Sequential Organ Failure Assessment (SOFA), albumin, creatinine, hemoglobin, white blood cell count, surgery type (model 2 not included), alcohol abuse, tobacco use, cerebrovascular disease, use of benzodiazepines and mechanical ventilation.

**Supplementary Table 6** Association between frailty and primary/secondary outcomes analysis using the full dataset (n=1952).

|  | Non-frail group^#^ | Frail group | P-value |
| --- | --- | --- | --- |
|  |  | OR (95% CI) |  |
| Delirium^●^  Unadjusted  Adjusted | Reference  Reference | 2.26 (1.79, 2.84)  1.70 (1.30, 2.23) | <0.001*  <0.001* |
| Pressure injury^●^  Unadjusted  Adjusted | Reference  Reference | 2.53 (1.77, 3.60)  1.84 (1.23, 2.76) | <0.001*  0.003* |

Abbreviations: OR: odds ratios; CI: confidence intervals.

Note:

^#^In our study, frailty status was defined based on the total score of the Modified Frailty Index (MFI): a score greater than 3 indicated frailty, a score of 1-2 indicated pre-frailty, and a score of 0 indicated no frailty. For the purpose of this study, a binary classification of pre-frailty status was performed using the MFI. Patients classified as "pre-frail" and "non-frail" were collectively referred to as "non-frail" patients.

^●^Logistic regression models were used to calculate odds ratios (OR) with 95% confidence intervals (CI).

*Significant difference between older patients after cardiac surgery between two groups (p < 0.05).

Delirium was adjusted for age, sex, race, marital status, Acute Physiology Score III (APSIII), Sequential Organ Failure Assessment (SOFA), albumin, creatinine, hemoglobin, white blood cell, surgery type, alcohol abuse, tobacco use, cerebrovascular disease, use of benzodiazepines and mechanical ventilation;

Pressure injury was adjusted for age, sex, race, marital status, APSIII, SOFA, Braden score, and surgery type.

**Supplementary Table 7** Association between frailty and delirium sensitive analysis using different combinations of covariates.

| Outcomes^●^ | Group^#^ | Model 1 |  | Model 2 |  | Model 3 |  | Model 4 |  |
| --- | --- | --- | --- | --- | --- | --- | --- | --- | --- |
|  |  | Adjusted OR (95% CI) | P-value | Adjusted OR (95% CI) | P-value | Adjusted OR (95% CI) | P-value | Adjusted OR (95% CI) | P-value |
| **Dlirium** | Non-frail group | Reference |  | Reference |  | Reference |  | Reference |  |
|  | Frail group | 2.26 (1.80, 2.84) | <0.001* | 1.62 (1.26, 2.07) | <0.001* | 1.67 (1.28, 2.17) | <0.001* | 1.55 (1.10, 2.19) | 0.012* |

Abbreviations: OR: odds ratios; CI: confidence intervals.

Note:

^#^In our study, frailty status was defined based on the total score of the Modified Frailty Index (MFI): a score greater than 3 indicated frailty, a score of 1-2 indicated pre-frailty, and a score of 0 indicated no frailty. For the purpose of this study, a binary classification of pre-frailty status was performed using the MFI. Patients classified as "pre-frail" and "non-frail" were collectively referred to as "non-frail" patients.

^●^Logistic regression models were used to calculate odds ratios (OR) with 95% confidence intervals (CI).

*Significant difference between older patients after cardiac surgery between two groups (p < 0.05).

Model 1 includes age, sex, race and marital status.

Model 2 builds on Model 1 by adding disease severity scores (SOFA and APSIII), and laboratory parameters (creatinine, albumin, hemoglobin and white blood cells).

Model 3 extends Model 2 by including type of surgery, treatment and medication (mechanical ventilation and use of benzodiazepines), and lifestyle (tobacco and alcohol abuse).

Model 4 extends Model 3 by including comorbidities such as myocardial infarct, congestive heart failure, peripheral vascular disease, chronic pulmonary disease, diabetes, renal disease and hypertension.

**Supplementary Figure 1** The missing rate of study variables.


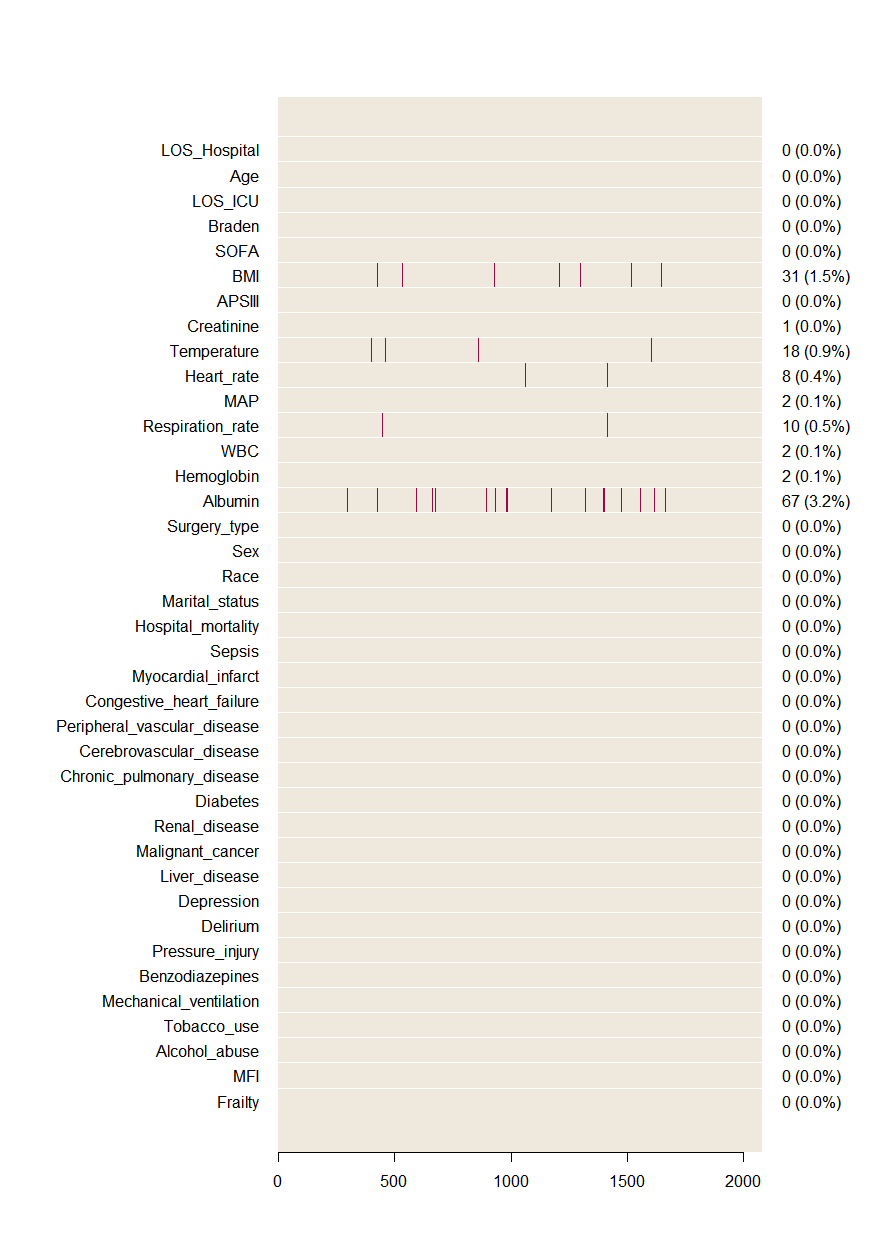


Abbreviations: ICU: Intensive Care Units; LOS: Length of Stay; SOFA: Sequential Organ Failure Assessment; APSIII: Acute Physiology Score III; BMI: Body Mass Index; MAP: Mean Arterial Pressure; WBC: White Blood Cell count; MFI: Modified Frailty Index.

Note: Figure showed features missing rate; One red block represented one missing value for each feature.

**Supplementary Figure 2** Restricted cubic spline regression was used to analyze the potential association between modified frailty index scores and risk of delirium in older patients after cardiac surgery.

**
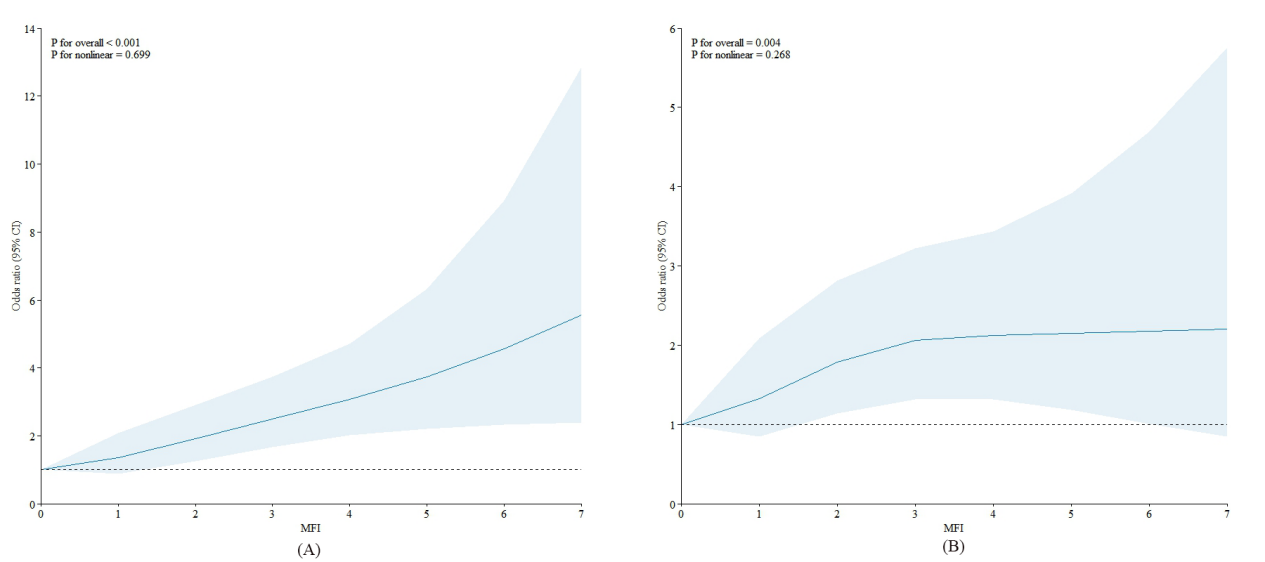
**

**(A)** Model I was an unadjusted model, **(B)** Model II was adjusted for age, sex, race, marital status, Acute Physiology Score III (APSIII), Sequential Organ Failure Assessment (SOFA), albumin, creatinine, hemoglobin, white blood cell, surgery type, alcohol abuse, tobacco use, cerebrovascular disease, use of benzodiazepines and mechanical ventilation.

Abbreviations: MFI, modified frailty index

**Supplementary Figure 3** Standardized mean differences before and after propensity score matching.

**
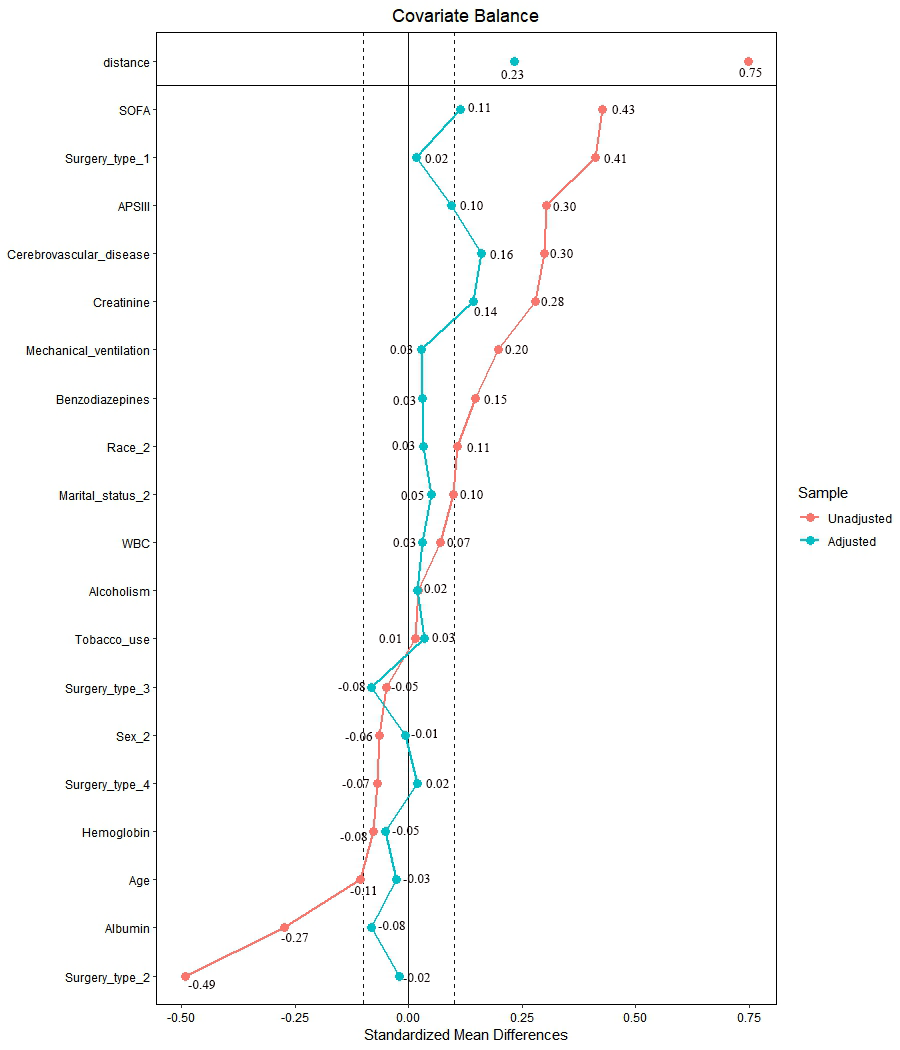
**

Abbreviations: SOFA: Sequential Organ Failure Assessment; APSIII: Acute Physiology Score III.
